# The association between neighbourhoods and educational achievement, a systematic review and meta-analysis

**DOI:** 10.1007/s10901-015-9460-7

**Published:** 2015-07-24

**Authors:** Jaap Nieuwenhuis, Pieter Hooimeijer

**Affiliations:** 1 Urban and Regional Research Centre Utrecht (URU), Faculty of Geosciences, Utrecht University, P.O. box 80115, 3508TC Utrecht, The Netherlands; 2 OTB - Research for the Built Environment, Faculty of Architecture and the Built Environment, Delft University of Technology, Delft, The Netherlands

**Keywords:** Education, Gender, Meta-analysis, Neighbourhood effects, Parental characteristics, Schools, Systematic review

## Abstract

Many studies have examined the effects of neighbourhoods on educational outcomes. The results of these studies are often conflicting, even if the same independent variables (such as poverty, educational climate, social disorganisation, or ethnic composition) are used. A systematic meta-analysis may help to resolve this lack of external validity. We identified 5516 articles from which we selected 88 that met all of the inclusion criteria. Using meta-regression, we found that the relation between neighbourhoods and individual educational outcomes is a function of neighbourhood poverty, the neighbourhood’s educational climate, the proportion of ethnic/migrant groups, and social disorganisation in the neighbourhood. The variance in the findings from different studies can partly be explained by the sampling design and the type of model used in each study. More important is the use of control variables (school, family SES, and parenting variables) in explaining the variation in the strength of neighbourhood effects.

## Introduction

The past two decades have seen an ongoing increase in the number of studies that investigate whether and how the neighbourhood in which people reside affects their socio-economic opportunities in life, of which educational achievement is one example (Cheshire 2012; Galster 2008; van Ham et al. 2012, 2013). This subject has also gained attention from policy makers in both Europe and the USA, resulting in a variety of neighbourhood-based policies founded on the idea that neighbourhood characteristics have an impact on residents (Blasius et al. 2009). Regardless of this widespread attention, uncertainty still exists about how a neighbourhood influences its residents, although there is some degree of consensus that interactions amongst residents are an important neighbourhood characteristic that influences the individuals in the neighbourhood (Galster 2012; Jencks and Mayer 1990).

Researchers base their understanding of the workings of the neighbourhood on several social mechanisms (for an overview, see Galster 2012) and use these mechanism to define neighbourhood characteristics that are likely to be important explanatory features for educational outcomes. The four most commonly used characteristics are: neighbourhood poverty, the educational climate, the proportion of migrant/ethnic groups, and social disorganisation. Because these characteristics are assumed to be related to different mechanisms, and therefore operate in different ways, we will examine them separately. Below, we will describe how the four characteristics relate to different neighbourhood mechanisms.

One of the social mechanisms cited is contagion, which describes the extent to which residents are influenced by their neighbours’ behaviour and attitudes. When negative attitudes towards education abound in a neighbourhood, its residents will be more inclined to adopt similar attitudes (Friedrichs 1998; Friedrichs and Blasius 2005). To test this model, the educational climate of the neighbourhood is often assessed. Another mechanism that is related to contagion is collective socialisation, which describes the collective ability of residents to cope with the social problems in the neighbourhood by influencing the behaviour of neighbours who do not conform to certain norms. In neighbourhoods that show higher levels of social cohesion and willingness to intervene in undesirable situations, residents are better able to enforce certain norms (Sampson et al. 1997), e.g. pro-learning norms and norms that assert the importance of education to a person’s future opportunities.

For neighbourhoods with higher levels of ethnic heterogeneity or higher concentrations of poverty, conflict theory predicts more disorder. People establish their identity by categorising themselves and others as members of different groups (Tajfel 1982). In neighbourhoods that experience competition over scarce resources such as jobs or neighbourhood facilities, residents tend to perceive out-group members as a threat (LeVine and Campbell 1972; Putnam 2007), which can generate socially disorganised neighbourhoods with a higher likelihood of crime and violence (Morenoff et al. 2001; Shaw and McKay 1942). Adolescent residents in such disorderly neighbourhoods experience greater exposure to peer groups that engage in deviant behaviour and possess negative attitudes towards education. This phenomenon relates back to the contagion mechanism and collective socialisation because the presence of such behaviour and attitudes can lead to their adoption by other residents. Furthermore, given a certain level of neighbourhood disorder, there may be less social cohesion, which may create a situation in which residents are less able to control deviant behaviour or enforce positive norms related to education.

Several reviews have attempted to summarise the literature about neighbourhood effects on educational outcomes, providing insight into the importance of neighbourhoods, the mechanisms by which neighbourhoods exert their influence, and the methodologies that can be used in this field. However, these reviews were conducted for specific subsamples (Johnson 2010), do not quantify their results (Dietz 2002; Leventhal and Brooks-Gunn 2000), or are dated (Jencks and Mayer 1990). Despite their significant value, such studies cannot explain the great diversity of results found in this field. We address these gaps through a systematic quantitative overview of the literature that has studied the influence of neighbourhood characteristics on educational outcomes. The variation in effect sizes might potentially be explained by differences between the study designs employed across the research in this area. To further examine this question, we use a meta-regression approach to analyse 88 studies. In this approach, we take the coefficients of the neighbourhood variables from the original studies and use them as the dependent variable in a new regression. This strategy allows us to identify the overall effect sizes of the four neighbourhood characteristics. Furthermore, we develop hypotheses regarding a range of study characteristics and test how they influence the results of the studies in question.

## Hypotheses

In this section, we consider how nine study characteristics might influence the neighbourhood effect. We begin by considering the context in which each study was conducted; more specifically, we look at the difference between USA- and Europe-based studies. Second, we consider the composition of the sample in terms of gender and age. Finally, we formulate hypotheses regarding the use of control variables such as previous individual educational attainment, parental behaviour, school characteristics, and family SES.

### Level of segregation

In the meta-analysis, we included only developed countries. Hence, we expect some degree of comparability between countries; however, we also expect some differences. Because most of the studies were conducted in the USA or (less commonly) in Europe, it is logical to investigate the differences between them. Ethnic and socio-economic segregation is higher in the USA than in Europe, and the ethnically concentrated neighbourhoods in Europe are more mixed in terms of the country of origin of their inhabitants than are those of the USA, where more mono-ethnic communities can be found (Musterd 2005; Wacquant 2008). The poor in Europe are not as isolated as in the USA, and they may gain more from their closer proximity to middle-class citizens, whereas the US poor tend to be more isolated and lack connections with the middle class (Wilson 1987). For the US poor, this can generate feelings of misrecognition due to stigmatisation, frustration about being denied the rights enjoyed by more affluent members of society, and the absence of perceived future opportunities because of a lack of good role models who perform well in school (Ainsworth 2002; Honneth 1995). There has been some support for threshold effect theories in neighbourhood research, indicating that beyond a certain threshold, the detrimental effect of neighbourhoods increases drastically (Galster 2014; Quercia and Galster 2000). This finding implies that at high levels of segregation, neighbourhood effects are more pronounced. At the end of the spectrum, neighbourhoods are more highly segregated in the USA than in Europe. Thus, we expect the US research to find stronger neighbourhood effects because the slope becomes much steeper past the threshold.

### Sample gender composition

The neighbourhood seems to be a stronger predictor of boys’ behaviour than girls’, which may partly be due to the greater amount of time that boys spend in the neighbourhood relative to girls (Ensminger et al. 1996; Entwisle et al. 1994); boys have greater exposure to characteristics of the neighbourhood that may influence them. The difference between boys and girls may also be explained as a function of parental monitoring: because girls are often more closely monitored by parents (Kim et al. 1999), parental monitoring may buffer girls from detrimental neighbourhood effects, whereas for boys, the influence of parental monitoring on the strength of the neighbourhood effect may be much weaker (Flouri and Ereky-Stevens 2008).

Furthermore, boys have been found to exhibit higher levels of externalising behaviour (e.g. aggression and delinquency) (Loeber and Hay 1997), which is related to lower educational success (Carroll et al. 2009; Kulka et al. 1982; McCluskey et al. 2002). Neighbourhoods with more social control may reduce this problematic behaviour to some extent (Drukker et al. 2009). Given these arguments, we expect boys to exhibit a stronger neighbourhood effect than girls.

### Sample age composition

The literature on educational achievement contains studies that examine different age groups. The age composition of a sample might influence neighbourhood effects to some extent. Because adolescents spend significant amounts of time away from their homes, parents are less able to monitor them (Kerr et al. 2010). This may result in greater exposure to the influence of a neighbourhood than younger children experience, as parents are better able to monitor the behaviour of the latter. Therefore, we expect stronger neighbourhood effects for adolescents than for younger children.

### Individual previous attainment

Neighbourhood residents are not randomly distributed over neighbourhoods; rather, they often cluster within neighbourhoods based on characteristics including income and educational attainment. The neighbourhood effects identified by studies that do not consider relevant background characteristics may be a result of the clustering of youth with certain educational attainment within certain neighbourhoods. Therefore, we expect studies that consider previous individual educational attainment indicators to find weaker neighbourhood effects.

### Parenting

Parental behaviour is assumed to be one of the key factors in adolescent development and educational outcomes (Bronfenbrenner 1979). Research that considers parenting within the context of a neighbourhood shows that parents adapt their parenting behaviour to the conditions of the neighbourhood (Duncan and Raudenbush 1999; Furstenberg et al. 1999). In high-poverty neighbourhoods, parents perceive the neighbourhood as a potential negative influence on their children’s development (Galster and Santiago 2006). To shield their children from this negative influence, parents in such neighbourhoods may use more protective parenting strategies or restrict outside recreational activities to areas where they can exert more supervision (e.g. the backyard) and ensure a safer environment for their children (Fauth et al. 2007; Furstenberg et al. 1999; Valentine and McKendrick 1997). In neighbourhoods with higher ethnic diversity, the reasoning is similar: the presence of people of different ethnicities can increase anxiety and distrust (Bauman 1993; LeVine and Campbell 1972; Putnam 2007), possibly encouraging more protective parenting strategies that can be used to protect children from the influence of out-groups (Nieuwenhuis et al. 2013). In using stricter monitoring strategies, parents attempt to minimise the effect that deviant neighbourhood peers may have on their children, thus attempting to control the influences to which their children are exposed despite the challenges posed by the neighbourhood in which they live (Furstenberg et al 1999; Jarrett 1997).

As argued above, parenting strategies vary with the neighbourhoods in which families reside. Because parenting is likely to be related to the extent to which children are protected from detrimental neighbourhood influences, we expect the neighbourhood variable slope coefficient to be different when parenting is controlled for in a study. Because of the greater perceived threat of neighbourhood influences in poor neighbourhoods (Galster and Santiago 2006), parents in poor neighbourhoods are likely to make more of an effort to monitor their children than do parents in affluent neighbourhoods (Fauth et al. 2007; Furstenberg et al. 1999), thereby weakening the negative effect of the neighbourhood. If a study fails to control for parenting, the weakening effect of parenting on the neighbourhood effect should be reflected in the neighbourhood coefficient, decreasing its slope. Studies that do control for parenting should find a stronger neighbourhood coefficient because the weakening influence of parenting on the neighbourhood effect is reflected in the parenting coefficient. The same reasoning applies if parenting is held constant across poor and affluent neighbourhoods, but it is assumed that children in poor neighbourhoods benefit more from parenting as a form of protection from negative neighbourhood influence. Studies that do not control for parenting may find a weaker neighbourhood effect because the shielding effect of parenting detracts from the neighbourhood effect. Including the parenting variable makes the neighbourhood effect more pronounced, and the weakening effect of parenting is reflected in the coefficient of the parenting variable. The above reasoning leads us to expect that controlling for parenting will strengthen the negative neighbourhood coefficient.

### Schools

Various social contexts shape the educational development of adolescents. Neighbourhoods are one such context, and schools are another (Bronfenbrenner 1979). Previous research has investigated how school and neighbourhood effects are related to their effect on educational outcomes; however, a consensus has not been reached. Some studies find that neighbourhood effects disappear after schools are controlled for (Sykes and Musterd 2011), whereas others find that the same effects remain (Bowen and Bowen 1999) and still others find that the results depend on how the neighbourhood and school variables are measured (Owens 2010; Pong and Hao 2007). Furthermore, studies that have considered the within-neighbourhood variance of educational achievement find a decrease in such variance after controlling for the school context (Brännström 2008; Kauppinen 2008).

The task of disentangling the influence of schools from neighbourhood effects is not a straightforward one. Schools may be a pathway through which neighbourhood effects are expressed given that poor neighbourhoods often have poor schools that have difficulty attracting good teaching staff because of their lack of resources (Jencks and Mayer 1990; Wacquant 2008). In addition, the demographic composition of a neighbourhood is often represented in the school population because school choice may be restricted or influenced by school catchment areas, information about schools from parents’ local social networks, or the proximity of certain schools. The resulting overlap between the demographics of the neighbourhood and those of the school makes it difficult to ascribe influence to one of the two contexts in particular. However, in disadvantaged neighbourhoods, parents may choose to send their children to schools outside their own neighbourhoods, where the quality of the education is expected to be better and the student demographics to be less disadvantageous (Furstenberg et al. 1999; Pinkster and Fortuijn 2009). Also, in the Moving to Opportunity programme, it was found that, after moving, parents might send their children to a school near their old neighbourhood, because it might be closer to family, and parents might be more familiar with the neighbourhood (Sanbonmatsu et al. 2006). In a study of youth delinquency, it emerged that adolescents who spend time outside of their own neighbourhoods with peers from other neighbourhoods are not affected by their own neighbourhoods (Oberwittler 2007). This finding suggests that when school and neighbourhood contexts do not overlap and when adolescents have more opportunities in school to meet peers from outside their own neighbourhoods, the likelihood of their being affected by their neighbourhoods may be smaller.

Students from poor areas are expected to be more likely to attend poor-quality schools. If not properly controlled for, the negative influence of such schools on the educational opportunities of students compared with those enrolled at higher-quality schools may spuriously be assigned to the neighbourhood instead. However, if we consider schools as a component of the institutional mechanisms through which a neighbourhood influences its residents, then controlling for school characteristics might to some degree minimise the explanatory power of the neighbourhood characteristics. In either case, we expect that studies that control for school-related variables will find weaker neighbourhood effects.

### Family SES

Neighbourhood research is often hampered by endogeneity problems and omitted variable bias. The neighbourhood in which one lives is not fixed but is rather the result of economic and social constraints. The social composition of neighbourhoods is the result of sorting. Although it is impossible to determine the exact sorting process, including control variables that are likely to be related to that process will decrease the level of omitted variable bias (Dietz 2002). Family socio-economic status is likely to be related to family choices regarding neighbourhood residence; thus, including family SES will likely decrease the level of omitted variable bias and therefore change the magnitude of the neighbourhood coefficients.

Two scenarios are possible: that omitting relevant variables will bias the neighbourhood coefficient downward or upward. On the one hand, because of economic constraints, poor families are more likely to live in poor neighbourhoods than are rich families, so neighbourhood SES is a partial proxy for the variation in family SES (Jencks and Mayer 1990). Furthermore, poor parents are more likely to lack access to the cultural and economic resources that they require to help their children succeed in a school environment, which may lead their children to exhibit lower educational attainment (Coleman 1988; Lareau 2003; Portes and MacLeod 1996). Because poor educational outcomes and a poor neighbourhood in this example are both the result of low family SES, omitting family SES will yield a stronger neighbourhood coefficient (Duncan et al. 1997). On the other hand, parents with higher SES are better equipped to help their children achieve the competences that are required for high performance in school and are found to allocate more time to child-rearing than do lower class parents (Bianchi et al. 2006; McLanahan 2004). High family SES in this example would partly compensate for the detrimental influence of a poor neighbourhood. This expectation is similar to our expectation for parenting: when not controlled for, the weakening effect of family SES on the neighbourhood effect will render the neighbourhood coefficient weaker. Hence, we hypothesise that studies that control for family SES will find stronger neighbourhood coefficients.

Testing these hypotheses (see Table 1 for an overview) will help to explain the variation in the results of different studies and will indicate how researchers can obtain more robust results in neighbourhood research. Moreover, investigating how such results are influenced by the chosen study design provides meaningful insight into the mechanisms through which a neighbourhood may influence its residents, strengthening the external validity of the relevant theory.

**Table 1:** Hypotheses

	Neighbourhood effect
H1: USA versus Europe	+
H2: Male sample versus female sample	+
H3: Adolescents versus younger children	+
H4: Control for previous individual educational attainment	−
H5: Control for parenting	+
H6: Control for school-related variables	−
H7: Control for family SES	+/−

+ Denotes that the neighbourhood effect is expected to be stronger; − denotes that the neighbourhood effect is expected to be weaker

## Method

### Data

**Fig. 1:**
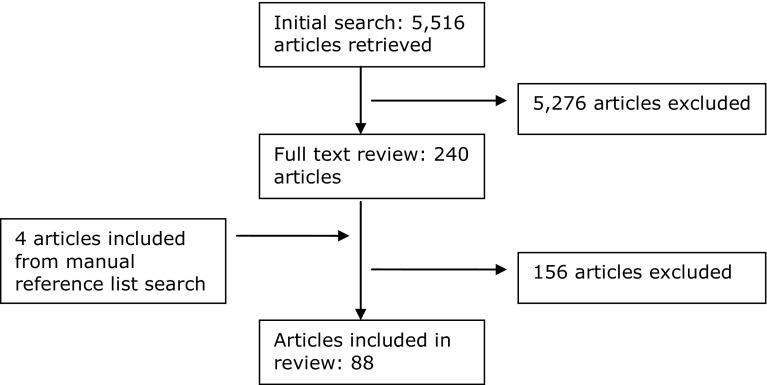
Flow chart of the literature search process

We identified relevant studies through a systematic search of Scopus that we conducted in October 2011. The search query included two themes: ‘neighbourhood’ and ‘education’. For both themes, the query required at least one of the search terms to be present in the title, abstract, or keywords of the study. The ‘neighbourhood’ theme included the following: neighb*rhood or ‘community characteristic*’ or ‘residen* characteristic*’ or ‘environment* characteristic*’ or ‘context* characteristic*’. ‘Education’ included the following: education* or school or grade* or drop*out or ‘drop out’ or academic*. The asterisk symbol is used to allow for every variant of a search term. The initial search yielded 5516 hits (see Fig. 1). Additionally, manual searches of the articles’ bibliographies were conducted to identify relevant studies that were not identified in the initial electronic search. This step yielded four additional studies. Filters were used to limit the results to social scientific studies in peer-reviewed journals. No language filter was used; however, because English search terms were used, non-English language studies were only included when an English abstract was provided. This process led to the inclusion of two non-English studies, one in Dutch and one in French.

The relevant studies were identified in two steps. First, the titles and abstracts of the studies were reviewed. This process yielded 244 potential candidate studies. In the second step, based on a full-text review, studies were included if they met the following criteria: (1) ‘educational achievement’ is the dependent variable (not included are those studies that use behavioural dependent variables such as truancy or expulsion from school); (2) the independent variables contain at least one neighbourhood characteristic; (3) (non-experimental) multivariate analysis is used; (4) a ‘neighbourhood’ is defined as ‘the neighbourhood in which the respondent lives/lived’ rather than as the area around the school that the respondent attends; (5) the sample used does not consist of preschoolers (as our goal was to accurately analyse educational outcomes rather than school-readiness); (6) the sample is from a developed country; (7) the study uses recent data, defined as data from 1960 to the present; and (8) the article provides information to obtain the coefficient and standard error. Of the 244 full-text review studies, 88 studies met all of the inclusion criteria (all included studies can be found in Appendix 1).

From each article, the following elements were recorded: sample size, sample age, sample gender composition, analysis type, operationalisation of ‘educational outcome’, coefficients and standard errors of the neighbourhood-level independent variables (34 in total), and information about the control variables. The dependent variable ‘educational outcome’ includes nine categories: (1) high school graduation rate; (2) high school dropout rate; (3) grades/test scores; (4) school performance (including teacher assessments and combinations of several categories); (5) grade retention; (6) years of education; (7) highest education; (8) college attendance; and (9) college graduation. When studies use high school dropout or grade retention as the outcome variable, the value of the dependent variable is inversed to orient the data in the same direction as the other educational outcome categories.

### Dependent variables

The dependent variables in the meta-regression are the unstandardised coefficients of the independent neighbourhood variables from the original studies. When odds are provided, we transformed these to log odds. This enabled us to calculate standard errors, which are necessary for our analyses (see the Analysis section). We constructed four dependent variables for the four analyses we conduct: poverty (*N* = 49), the educational climate (*N* = 17), the proportion of migrant/ethnic groups (*N* = 48), and social disorganisation (*N* = 47). The four variables are combinations of sets of predefined variables from the original studies. If a study contains one of the predefined variables, the value of the coefficient is included in the dependent variable. For studies that contain more than one of these variables, the coefficient of the variable with the highest absolute magnitude after weighting using the inverse of the standard error is included. Neighbourhood poverty is analysed using the following variables from the original studies: the proportion of the population with a low SES, the proportion of poor households, the proportion of rich households (inversed), the share of the population that is unemployed, institutional resources (inversed), the proportion of high-status residents (inversed), the share of homeowners (inversed), the proportion of single mothers, and the variables used in previous studies that combine some of these other variables. Educational climate is negatively coded and should be interpreted as indicating a poor educational climate. This category includes the proportion of high school dropouts; the share of high-educated individuals (inversed); peer grades (inversed); and the proportion of youth in school (inversed). The proportion of migrant/ethnic group variable takes into account both the proportion of migrant/ethnic groups and the proportion of Whites (inversed). Social disorganisation takes into account positive perceptions of the neighbourhood (inversed), social cohesion (inversed), social control (inversed), disorder/crime, poor physical conditions, residential stability (inversed), and population density.

### Covariates

The study characteristics are extracted to test their influence on the results. The location where the study was conducted is coded using three dummies: the USA, (Northern and Western) Europe, and other. The ‘other’ category includes Canada (4 studies), Australia (3), and Taiwan (1).

Two characteristics of the sample are included: age and gender. Sample age is coded using three dummies: 4–10, 11–20, and 21 years and older. Studies that contained samples that had overlap within these categories are included in the category that contained the largest part of their sample. Gender is also coded using three dummies: male, female, and mixed.

The analyses contain four dummies that measure the use of certain control variables in the original study: one for school-level control variables (e.g. private school, school SES, or student–teacher ratio); one for controls related to parenting behaviour of respondents’ parents (e.g. parental involvement in school, restrictive parenting, or supportive parenting); one for controls related to respondents’ family SES (e.g. family income, parents’ social class, or parents’ occupation status); and one for control variables that reflect previous individual educational attainment (e.g. prior test scores).

Sample size and the use of multilevel analysis are associated with more precise results. It is unclear whether a more precise neighbourhood effect is a weaker or stronger neighbourhood effect. However, including control variables for sample size and the use of multilevel will enable us to reveal whether the ‘true’ neighbourhood effect is weaker or stronger. We do not expect the effect of sample size to be linear; hence, we take the log of the sample size. For the use of multilevel modelling, we include a dummy.

Because educational outcomes are grouped into nine categories and are thus not operationalised in the same way in all studies, we include control dummies for this. Dependent on the distribution of the categories in a model, we include dummies for single categories or dummies for combinations of categories. All of the covariates are standardised. The descriptive statistics for the unstandardised variables for all four models can be found in Appendix 2 (Tables 6, 7, 8 and 9).^1^

### Analysis

We conducted the four analyses using random-effects meta-regression. The models use the coefficients of the independent neighbourhood variables from the original studies as the dependent variables. The coefficients are estimated via weighted least squares using the inverse of the between-study variance (*τ* ^2^) and the standard error ($\sigma_{i}^{2}$) of the estimated effect in the original study *i* as the weight $(1/(\sigma_{i}^{2} + \tau^{2} ))$ (Harbord and Higgins 2008). Because of this weighting process, more precise studies (i.e. studies with smaller standard errors) have more influence in the analysis. The meta-regression also indicates the between-study variance (*τ* ^2^) and the proportion of the residual variation that can potentially be explained by study-level covariates ($I_{\text{res}}^{2}$).

Several of the studies contain analyses of subgroups: for example, analyses of males and females or of an ethnic sample and a native sample. Using studies or subgroups as the unit of analysis yields no difference with regard to the computed summary effect and variance. However, it does yield a different level of between-study variance (Borenstein et al. 2009). We expect the effects to differ for the different groups; therefore, we use subgroups as the unit of analysis, effectively computing the between-study variance based on the subgroups. This results in *N*’s of 94, 17, 48, and 47.

## Results

Table 2 shows the results of the meta-regression for neighbourhood poverty. Looking first at the intercept, we see a clear negative result of neighbourhood poverty on educational achievement, even after taking into account a large range of study characteristics. The positive coefficient of ‘other location’ indicates that this neighbourhood effect is smaller in Australia and Canada (the study from Taiwan is not included in this analysis) than in Europe. The statistical and sample-specific covariates do not seem to influence the results, although the log of sample size has a marginally significant negative effect. The results do seem to differ when different educational outcomes are investigated: studies that examine school performance, college education, or years of education yield weaker results than do studies that examine grades or test scores.

Looking at the use of specific control variables, we see that controlling for school-related variables decreases magnitude of the effect of the neighbourhood. Controlling for parenting increases the magnitude of the neighbourhood poverty coefficient, as does controlling for family SES. Studies that control for previous individual educational achievement do not seem to find results that are different from those of studies that do not control for it.

**Table 2:** Meta-regression for neighbourhood poverty (*N* = 94)

	Coef.	SE	*t*
Location (ref.: Europe)			
Location: USA	.021	.040	.53
Location: other	.066*	.030	2.24
Sample gender (ref.: female)			
Sample gender: male	.001	.037	.02
Sample gender: mixed	−.038	.049	−.78
Sample age (ref.: 11–20 years)			
Sample age: 4–10 years	.039	.046	.86
Sample age: 21+ years	.003	.042	.07
Previous educational attainment control variables	.042	.037	1.16
Parenting control variables	−.139**	.049	−2.84
School-level control variables	.075*	.038	1.99
Family SES control variables	−.094^†^	.049	−1.92
Sample size (log)	−.065^†^	.039	−1.68
Use of multilevel	.049	.042	1.19
Educ. outcome (ref.: grades/test scores)			
Educ. outcome: high school graduation and high school dropout	.050	.050	1.00
Educ. outcome: school performance and grade retention	.094*	.040	2.33
Educ. outcome: years of education; highest education; college attendance and c. graduation	.105*	.051	2.07
Intercept	−.159**	.037	−4.31
Between-study variance (*τ* ^2^)	.02477		
Proportion residual variation $\left( {I_{\text{res}}^{2} } \right)$	.9142		

The *N* is based on analyses of subgroups

^†^ *p* < .10; * *p* < .05; ** *p* < .01

The results of the meta-regression for poor educational climate in the neighbourhood are shown in Table 3. The intercept shows a negative association between a poor educational climate and educational achievement. This association does not seem to be weaker when high school graduation is used as educational outcome variables, compared with grades/test scores, school performance, and grade retention. Comparing the US and European studies indicates that the American studies yielded much stronger negatives than the European ones. Furthermore, a larger sample size increases the magnitude of the neighbourhood coefficient. Studies that use samples with respondents who are 21 years or older find weaker effects than do studies that use samples composed of 11- to 20-year-olds. Controlling for school-related variables increases the strength of the neighbourhood coefficient.

**Table 3:** Meta-regression for poor educational climate (*N* = 17)

	Coef.	SE	*t*
Location (ref.: Europe and other)			
Location: USA	−.264*	.096	−2.76
Sample age (ref.: 11–20 years)			
Sample age: 21+ years	.192*	.072	2.66
School-level control variables	−.320*	.103	−3.11
Sample size (log)	−.274*	.089	−3.09
Use of multilevel	.095	.071	1.34
Educ. outcome (ref.: grades/test scores; school performance and grade retention)			
Educ. outcome: high school graduation	.127*	.047	2.68
Educ. outcome: years of education; highest education and college attendance	.099	.057	1.74
Intercept	−.503**	.134	−3.76
Between-study variance (*τ* ^2^)	.01465		
Proportion residual variation $\left( {I_{\text{res}}^{2} } \right)$	.8773		

The *N* is based on analyses of subgroups

^†^ *p* < .10; * *p* < .05; ** *p* < .01

The meta-regression for the proportion of migrant/ethnic groups in the neighbourhood (Table 4) yields a negative intercept, indicating that individuals in neighbourhoods with higher proportions of migrant or ethnic groups achieve less with regard to their education. This result does not seem to change when different categories of educational outcomes are used. The use of multilevel analysis increases the strength of the negative neighbourhood coefficient. However, because this is the only model in which we find a significant effect, we cannot say whether the use of multilevel analysis systematically yields weaker or stronger neighbourhood effects. Additionally, studies that control for parenting find a stronger negative effect.

**Table 4:** Meta-regression for the proportion of migrant/ethnic groups (*N* = 48)

	Coef.	SE	*t*
Location (ref.: Europe and other)			
Location: USA	−.003	.005	−.50
Sample gender (ref.: female)			
Sample gender: male	−.002	.001	−1.09
Sample gender: mixed	.002	.004	.52
Sample age (ref.: 11–20 years)			
Sample age: 4–10 years	.006	.023	.25
Sample age: 21+ years	−.003	.004	−.57
Previous educational attainment control variables	−.028	.021	−1.37
Parenting control variables	−.115**	.031	−3.72
School-level control variables	.000	.005	−.04
Sample size (log)	.000	.004	−.06
Use of multilevel	−.010**	.003	−3.66
Educ. outcome (ref.: grades/test scores)			
Educ. outcome: high school graduation and high school dropout	−.024	.019	−1.27
Educ. outcome: school performance and grade retention	−.006	.013	−.47
Educ. outcome: years of education and college attendance	−.024	.019	−1.27
Intercept	−.035**	.013	−2.74
Between-study variance (*τ* ^2^)	.00000		
Proportion residual variation $\left( {I_{\text{res}}^{2} } \right)$	.5635		

The *N* is based on analyses of subgroups

^†^ *p* < .10; * *p* < .05; ** *p* < .01

The meta-analysis of neighbourhood social disorganisation is shown in Table 5, where we find a negative overall effect on educational achievement, a result that is much smaller when the sample size increases. In addition, controlling for family SES seems to decrease the size of the coefficient. Other covariates do not influence the neighbourhood coefficients.

**Table 5:** Meta-regression for social disorganisation (*N* = 47)

	Coef.	SE	*t*
Location (ref.: Europe and other)			
Location: USA	−.006	.020	−.28
Sample age (ref.: 11–20 years)			
Sample age: 4–10 years	.019	.021	.89
Sample age: 21+ years	.036	.029	1.23
Previous educational attainment control variables	.002	.019	.08
Parenting control variables	−.013	.023	−.55
School-level control variables	−.009	.022	−.41
Family SES control variables	.046*	.019	2.45
Sample size (log)	.047^†^	.023	2.02
Use of multilevel	−.008	.020	−.38
Educ. outcome (ref.: everything else)			
Educ. outcome: grades/test scores	.016	.041	.38
Intercept	−.073*	.028	−2.59
Between-study variance (*τ* ^2^)	.00053		
Proportion residual variation $\left( {I_{\text{res}}^{2} } \right)$	.7164		

The *N* is based on analyses of subgroups

^†^ *p* < .10; * *p* < .05; ** *p* < .01

## Conclusion and discussion

This meta-analysis reviews the quantitative research that has been conducted on the association between neighbourhoods and individual educational outcomes. We found that individual educational outcomes are significantly associated with all four neighbourhood characteristics we studied: neighbourhood poverty, a poor educational climate, the proportion of ethnic/migrant groups, and social disorganisation.

The main purpose of this study was to test whether heterogeneity in the findings of neighbourhood effects studies can be explained by the designs of the different studies. Beginning by examining the institutional environment, we find some support for the hypothesis that neighbourhood effects in general differ across different environments. For the specific neighbourhood variable ‘poor educational climate’, we do find a significant difference between the USA and Europe; much stronger negative neighbourhood effects are found in the USA. Additionally, in the other three models, the sign of the coefficients also suggests that the US findings are stronger, but the coefficients are not significant. This result suggests that the higher concentration of disadvantaged groups in the USA leads to a steeper neighbourhood effect.

The composition of the sample with regard to gender was argued to influence the strength of the neighbourhood effect because of boys’ higher exposure to their neighbourhoods. However, we find no proof that this is the case for any of the four studied neighbourhood effects. We also expected to find differences between different age groups; however, we do not find strong support for the hypothesis that different neighbourhood effects are found across age groups. Only the model for poor educational climate shows that when studies use samples composed of 21-year-olds or older individuals, they find weaker negative effects than when samples of 11- to 20-year-olds are used. This finding indicates that the influence of the neighbourhood is stronger for adolescents than for young adults. However, because only one of the four models finds a difference between the age groups, this finding should not be interpreted as a strong claim.

We coded four types of control variables that studies could have used: previous individual educational attainment, parenting behaviour, school-level control variables, and family SES. Unexpectedly, we do not find that controlling for previous educational attainment has a significant influence on the strength of the neighbourhood effect; there is no support for the claim that neighbourhood effects are found because this type of heterogeneity within the sample is not controlled for. Nevertheless, looking only at the direction of the results in the models for poverty and social disorganisation, we can see that previous attainment decreases the magnitude of the neighbourhood coefficients, as predicted. However, because adolescents often have little influence over their families’ choice of neighbourhoods, such heterogeneity might be better reflected by the parents.

A number of studies control for parenting behaviour in their models. We hypothesised that in neighbourhoods with high levels of poverty or ethnic heterogeneity, either parents parent more or youths benefit more from parenting than in low-poverty neighbourhoods. Therefore, when parenting is omitted from the model, the shielding effect of parenting on the neighbourhood’s influence on educational achievement will be incorporated in the neighbourhood coefficient, rendering it weaker. Conversely, when it is included, the neighbourhood effect will be stronger. This hypothesis is supported for poverty, for the proportion of migrant/ethnic groups in the neighbourhood, and (albeit insignificant) for social disorganisation; in all three cases, a stronger negative neighbourhood effect is found when parenting is controlled for. Moreover, in the model for the proportion of migrant/ethnic groups, the coefficient of controlling for parenting has a considerably larger magnitude than the coefficients of the other covariates, suggesting that it might be difficult for studies to determine the effect of the presence of migrant/ethnic groups within a neighbourhood when they do not take parenting into account in their models. Different explanations for these results are possible. Neighbourhood effects could be mediated by parenting, or there could be an interaction effect between neighbourhood characteristics and the benefits gained from parenting. Because parenting includes different dimensions (e.g. support and control), it might be fruitful to consider how neighbourhoods affect different dimensions of parenting and, consequently, how these different dimensions relate to the relationship between the neighbourhood and educational outcomes (e.g. Nieuwenhuis et al. 2013). Parental control may increase when neighbourhood poverty increases because parents want to protect their children from detrimental neighbourhood effects. However, parental support may decrease because neighbourhood poverty and disorder may increase parental stress, which is associated with less supportive parenting (Downey and Coyne 1990; Kohen et al. 2008). Different parenting behaviours may have different effects on educational outcomes, which can generate interesting research questions about the relationship between neighbourhoods, parenting, and educational outcomes.

Controlling for school-related variables was expected to weaken the neighbourhood effect, either because school effects are ascribed to the neighbourhood when school-related variables are not controlled for or because neighbourhood effects might disappear due to over-controlling for school characteristics. This supposition is supported in the model for neighbourhood poverty, where controlling for school variables weakens the neighbourhood variable. However, it must be noted that different policies exist across countries with reference to school catchment areas and school choice—and that as a result, schools are not necessarily located in the neighbourhood in which the students live. Given this variance, we cannot be certain that the same mechanism is present in all of the studies examined here. Furthermore, contrary to our expectations, in the model for poor educational climate, the negative neighbourhood coefficient is strengthened when school variables are controlled for. The same relationship is suggested when we examine the sign of the insignificant covariates for the school in the models for migrant/ethnic groups and social disorganisation. One possible explanation for this finding could be that good schools compensate for the detrimental effects of a bad neighbourhood. Therefore, when school-related variables are controlled for, the estimation of the neighbourhood effect is not influenced by the differences between the schools that the students attend, and a stronger neighbourhood effect results. Because of the contradictory findings of the models for neighbourhood poverty and poor educational climate, it appears that two mechanisms are present that work in opposite directions. Future research should attempt to determine when each mechanism is more important.

We suggest two opposing scenarios that indicate how the neighbourhood effect may change when family SES is not controlled for. One scenario involves downward bias and the other upward bias. We found support for both scenarios. First, in the model for neighbourhood poverty, controlling for family SES yields stronger negative neighbourhood effects. This finding suggests that differences in family SES within neighbourhoods lead to the underestimation of the neighbourhood effect when family SES is omitted from the model. Second, in the model for social disorganisation, controlling for family SES leads to weaker negative neighbourhood effects. This finding suggests that both educational achievement and the neighbourhood in which people live are the result of family SES. When SES is omitted, spurious neighbourhood effects are found that are actually caused by family SES. Given that both scenarios are supported by the data, it would be interesting to more deeply consider this question to determine the dynamic between family SES, neighbourhood characteristics, and individual outcomes.

The models still exhibit residual variation, which can potentially be explained by additional study-level covariates. First, the mechanisms that explain the neighbourhood effects are often based on interactions between people; therefore, the assumption is that social networks play a significant role. The neighbourhood delineation that best captures an individual social network is a contested issue; additionally, networks outside of the neighbourhood are likely to also influence resident outcomes. Second, studies could employ more sophisticated statistical models or longitudinal designs to attempt to overcome selection effect bias, which might yield different results than have been obtained by studies that have not used these tools. However, because such approaches are quite novel and diverse, there is not enough variation to capture these elements in workable covariates. Third, different results might be obtained from studies that use linear and nonlinear neighbourhood variables. However, because different nonlinear studies are not equally operationalised and because it is difficult to predict how nonlinear variables would behave in meta-regression analyses, including nonlinear variables would pose great difficulties. Fourth, different findings could result from differences in sample composition (e.g. with regard to income, ethnicity, or personality) because some groups might be more vulnerable to the influence of context (see e.g. Galster et al. 2010; Nieuwenhuis et al. 2015). We did not include these considerations due to the high number of missing values for income, ethnic background, or personality within the sample. Lastly, for the purpose of obtaining big enough samples, we collapsed all neighbourhood characteristics into four categories. Even though these categories are informed by the literature, the neighbourhood characteristics within categories might still differ to some extent. This can potentially also increase the residual variance of the models. These differences between studies are likely to partially explain the residual variation; thus, further examining these issues is likely to provide additional insight in the variation between the results of different studies; however, such efforts lie beyond the scope of this study.

The main conclusions of this review are as follows. First, our analyses of the current literature have shown that at least four neighbourhood characteristics do influence educational outcomes. Second, study-level characteristics seem to have a substantial influence on the neighbourhood effects that are found in different studies. Most importantly, it is necessary to add the right control variables to the model to avoid overestimating or underestimating neighbourhood effects. Also, depending on which neighbourhood characteristic is studied, we found differences in how model specifications influence the magnitude of the neighbourhood effect. Different neighbourhood characteristics imply different mechanisms for how the neighbourhood might influence its residents, so careful thought is required about how the neighbourhood might operate and which factors might influence the possible mechanisms. Besides that, in this study we have focussed on educational outcomes. The field of neighbourhood studies is broad, and outcomes such as health, income, or delinquency might require different considerations. In sum, close attention to how studies are designed is warranted, and this meta-analysis provides some clues about what requires attention.

## Appendix 1: Studies included in the meta-analysis

- Aaronson, D. (1998) Using sibling data to estimate the impact of neighborhoods on children’s educational outcomes, *Journal of Human Resources*, 33(4), pp. 915–946.
- Ainsworth, J. W. (2002) Why does it take a village? The mediation of neighborhood effects on educational achievement, *Social Forces*, 81(1), pp. 117–152.
- Ainsworth, J. W. (2010) Does the race of neighborhood role models matter? Collective socialization effects on educational achievement, *Urban Education*, 45(4), pp. 401–423.
- Andersson, E. and Subramanian, S. V. (2006) Explorations of neighbourhood and educational outcomes for young Swedes, *Urban Studies*, 43(11), pp. 2013–2025.
- Andreias, L., Borawski, E., Schluchter, M., Taylor, H. G., Klein, N. and Hack, M. (2010) Neighborhood influences on the academic achievement of extremely low birth weight children, *Journal of Pediatric Psychology*, 35(3), pp. 275–283.
- Åslund, O., Edin, P.-A., Fredriksson, P. and Grönqvist, H. (2011) Peers, neighborhoods, and immigrant student achievement: Evidence from a placement policy,. *American Economic Journal: Applied Economics*, 3(2), pp. 67–95.
- Benson, J. and Borman, G. D. (2010) Family, neighborhood, and school settings across seasons: When do socioeconomic context and racial composition matter for the reading achievement growth of young children? *Teachers College Record*, 112(5), pp. 1338–1390.
- Bowen, N. K. and Bowen, G. L. (1999) Effects of crime and violence in neighborhoods and schools on the school behavior and performance of adolescents, *Journal of Adolescent Research*, 14(3), pp. 319–342.
- Boyle, M. H., Georgiades, K., Racine, Y. and Mustard, C. (2007) Neighborhood and family influences on educational attainment: Results from the Ontario Child Health Study Follow-Up 2001, *Child Development*, 78(1), pp. 168–189.
- Bramley, G. and Karley, N. K. (2007) Homeownership, poverty and educational achievement: School effects as neighbourhood effects, *Housing Studies*, 22(5), pp. 693–721.
- Brännström, L. (2008) Making their mark: the effects of neighbourhood and upper secondary school on educational achievement, *European Sociological Review*, 24(4), pp. 463–478.
- Brooks-Gunn, J., Duncan, G. J., Klebanov, P. K. and Sealand, N. (1993) Do Neighborhoods Influence Child and Adolescent Development? *American Journal of Sociology*, 99(2), pp. 353–395.
- Bygren, M. and Szulkin, R. (2010) Ethnic environment during childhood and the educational attainment of immigrant children in Sweden, *Social Forces*, 88(3), pp. 1305–1329.
- Byrd, C. M. and Chavous, T. M. (2009) Racial identity and academic achievement in the neighborhood context: A multilevel analysis, *Journal of Youth and Adolescence*, 38(4), pp. 544–559.
- Cardak, B. A. and McDonald, J. T. (2004) Neighbourhood effects, preference heterogeneity and immigrant educational attainment, *Applied Economics*, 36(6), pp. 559–572.
- Catsambis, S. and Beveridge, A. A. (2001) Does neighborhood matter? Family, neighborhood, and school influences on eighth-grade mathematics achievement, *Sociological Focus*, 34(4), pp. 435–457.
- Charles, C. Z., Dinwiddie, G. and Massey, D. S. (2004) The continuing consequences of segregation: Family stress and college academic performance, *Social Science Quarterly*, 85(5), pp. 1353–1373.
- Chase-Lansdale, P. L. and Gordon, R. A. (1996) Economic hardship and the development of five- and six-year-olds: Neighborhood and regional perspectives, *Child Development*, 67, pp. 3338–3367.
- Connell, J. P., Halpern-Felsher, B. L., Clifford, E., Crichlow, W. and Usinger, P. (1995) Hanging in there: Behavioral, psychological, and contextual factors affecting whether African American adolescents stay in high school, *Journal of Adolescent Research*, 10(1), pp. 41–63.
- Connell, J. P., Spencer, M. B. and Aber, J. L. (1994) Educational risk and resilience in African-American youth: context, self, action, and outcomes in school, *Child development*, 65, pp. 493–506.
- Coombes, M. and Raybould, S. (1997) Modelling the influence of individual and spatial factors underlying variations in the levels of secondary school examination results, *Environment and Planning A*, 29(4), pp. 641–658.
- Crane, J. (1999) The epidemic theory of ghettos and neighborhood effects on dropping out and teenage childbearing, *American Journal of Sociology*, 96(5), pp. 1226–1259.
- Crowder, K. and South, S. J. (2003) Neighborhood distress and school dropout: The variable significance of community context, *Social Science Research*, 32(4), pp. 659–698.
- Crowder, K. and South, S. J. (2011) Spatial and temporal dimensions of neighborhood effects on high school graduation, *Social Science Research*, 40(1), pp. 87–106.
- Crowder, K. and Teachman, J. (2004) Do residential conditions explain the relationship between living arrangements and adolescent behavior? *Journal of Marriage and Family*, 66(3), pp. 721–738.
- Datcher, L. (1982) Effects of community and family background on achievement, *Review of Economics and Statistics*, 64(1), pp. 32–41.
- Dearing, E. (2004) The developmental implications of restrictive and supportive parenting across neighborhoods and ethnicities: Exceptions are the rule, *Journal of Applied Developmental Psychology*, 25(5), pp. 555–575.
- Duncan, G. J. (1994) Families and neighbors as sources of disadvantage in the schooling decisions of white and black adolescents, *American Journal of Education*, 103(1), pp. 20–53.
- Drukker, M., Feron, F. J. M., Mengelers, R. and Van Os, J. (2009) Neighborhood socioeconomic and social factors and school achievement in boys and girls, *Journal of Early Adolescence*, 29(2), pp. 285–306.
- Dupere, V., Leventhal, T., Crosnoe, R. and Dion, E. (2010) Understanding the positive role of neighborhood socioeconomic advantage in achievement: The contribution of the home, child care, and school environments, *Developmental Psychology*, 46(5), pp. 1227–1244.
- Eamon, M. K. (2005) Social-demographic, school, neighborhood, and parenting influences on the academic achievement of Latino young adolescents, *Journal of Youth and Adolescence*, 34(2), pp. 163–174.
- Ensminger, M. E., Lamkim, R. P. and Jacobson, N. (1996) School leaving: A longitudinal perspective including neighborhood effects, *Child Development*, 67, pp. 2400–2416.
- Flores, R. J. O. (2002) An examination of neighborhood effects on patterns of high school attrition among Puerto Rican youth in the New York Metropolitan Area, *Journal of Hispanic Higher Education*, 1(1), pp. 69–87.
- Flouri, E. and Ereky-Stevens, K. (2008) Urban neighbourhood quality and school leaving age: Gender differences and some hypotheses, *Oxford Review of Education*, 34(2), pp. 203–216.
- Foster, E. M. and McLanahan, S. (1996) An illustration of the use of instrumental variables: Do neighborhood conditions affect a young person’s chance of finishing high school? *Psychological Methods*, 1(3), pp. 249–260.
- Galster, G., Marcotte, D. E., Mandell, M., Wolman, H. and Augustine, N. (2007) The influence of neighborhood poverty during childhood on fertility, education, and earnings outcomes, *Housing Studies*, 22(5), pp. 723–751.
- Garner, C. L. and Raudenbush, S.W. (1991) Neighborhood effects on educational attainment: A multilevel analysis, *Sociology of Education*, 64(4), pp. 251–262.
- Georgiades, K., Boyle, M. H. and Duku, E. (2007) Contextual influences on children’s mental health and school performance: The moderating effects of family immigrant status, *Child Development*, 78(5), pp. 1572–1591.
- Ginther, D., Haveman, R. and Wolfe, B. (2000) Neighborhood attributes as determinants of children’s outcomes: How robust are the relationships? *Journal of Human Resources*, 35(4), pp. 603–642.
- Goldsmith, P. R. (2009) Schools or neighborhoods or both? Race and ethnic segregation and educational attainment, *Social Forces*, 87(4), pp. 1913–1942.
- Gonzales, N. A., Cauce, A.M., Friedman, R.J. and Mason, C.A. (1996) Family, peer, and neighborhood influences on academic achievement among African-American adolescents: One-year prospective effects, *American Journal of Community Psychology*, 24(3), pp. 365–387.
- Goux, D. and Maurin, E. (2005) Composition sociale du voisinage et échec scolaire. Une évaluation sur données françaises [The effects of neighbourhoods on early performance at school in France], *Revue Economique*, 56(2), pp. 349–362.
- Goux, D. and Maurin, E. (2007) Close neighbours matter: Neighbourhood effects on early performance at school, *Economic Journal*, 117(523), pp. 1193–1215.
- Greenman, E., Bodovski, K. and Reed, K. (2011) Neighborhood characteristics, parental practices and children’s math achievement in elementary school, *Social Science Research*, 40(5), pp. 1434–1444.
- Han, W.-J. (2008) Adolescents of New York City immigrant families, *Children and Youth Services Review*, 30(10), pp. 1144–1158.
- Harding, D. J. (2003) Counterfactual models of neighborhood effects: The effect of neighborhood poverty on dropping out and teenage pregnancy, *American Journal of Sociology*, 109(3), pp. 676–719.
- Harding, D. J. (2011) Rethinking the cultural context of schooling decisions in disadvantaged neighborhoods: From deviant subculture to cultural heterogeneity, *Sociology of Education*, 84(4), pp. 322–339.
- Henry, C. S., Merten, M. J., Plunkett, S. W. and Sands, T. (2008) Neighborhood, parenting, and adolescent factors and academic achievement in Latino adolescents from immigrant families, *Family Relations*, 57(5), pp. 579–590.
- Herman, M. R. (2009) The Black-White-other achievement gap: testing theories of academic performance among multiracial and monoracial adolescents, *Sociology of Education*, 82(1), pp. 20–46.
- Ioannides, Y. M. (2003) Empirical nonlinearities and neighbourhood effects in the intergenerational transmission of human capital, *Applied Economics Letters*, 10(9), pp. 535-539.
- Joo, M. (2010) Long-term effects of Head Start on academic and school outcomes of children in persistent poverty: Girls vs. boys, *Children and Youth Services Review*, 32(6), pp. 807–814.
- Kauppinen, T. M. (2007) Neighborhood effects in a European city: Secondary education of young people in Helsinki, *Social Science Research*, 36(1), pp. 421–444.
- Kauppinen, T. M. (2008) Schools as mediators of neighbourhood effects on choice between vocational and academic tracks of secondary education in Helsinki, *European Sociological Review*, 24(3), pp. 379–391.
- Kowaleski-Jones, L., Dunifon, R. and Ream, G. (2006) Community contributions to scholastic success, *Journal of Community Psychology*, 34(3), pp. 343–362.
- Lauen, D. L. (2009) To choose or not to choose: High school choice and graduation in Chicago, *Educational Evaluation and Policy Analysis*, 31(3), pp. 179–199.
- Leckie, G. (2009) The complexity of school and neighbourhood effects and movements of pupils on school differences in models of educational achievement, *Journal of the Royal Statistical Society. Series A: Statistics in Society*, 172(3), pp. 537–554.
- Lindsay, C. A. (2011) All middle-class families are not created equal: Explaining the contexts that Black and White families face and the implications for adolescent achievement, *Social Science Quarterly*, 20(3), pp. 201–209.
- Lloyd, J. E. V., Li, L. and Hertzman, C. (2010) Early experiences matter: Lasting effect of concentrated disadvantage on children’s language and cognitive outcomes, *Health and Place*, 16(2), pp. 371–380.
- López Turley, R. N. (2003) When do neighborhoods matter? The role of race and neighborhood peers, *Social Science Research*, 32, pp. 61–79.
- Madyun, N. and Lee, M. (2008) Community influences on E/BD student achievement, *Education and Urban Society*, 40(3), pp. 307–328.
- McCulloch, A. and Joshi, H. E. (2001) Neighbourhood and family influences on the cognitive ability of children in the British National Child Development Study, *Social Science and Medicine*, 53(5), pp. 579–591.
- McWayne, C. M., McDermott, P. A., Fantuzzo, J. W. and Culhane, D. P. (2007) Employing community data to investigate social and structural dimensions of urban neighborhoods: An early childhood education example, *American Journal of Community Psychology*, 39, pp. 47–60.
- Milam, A. J., Furr-Holden, C. D. M. and Leaf, P. J. (2010) Perceived school and neighborhood safety, neighborhood violence and academic achievement in urban school children, *Urban Review*, 42(5), pp. 458–467.
- Mohanty, L. L. and Raut, L. K. (2009) Home ownership and school outcomes of children: Evidence from the PSID child development supplement, *American Journal of Economics and Sociology*, 68(2), pp. 465–489.
- Mollenkopf, J. and Champeny, A. (2009) The neighbourhood context for second-generation education and labour market outcomes in New York, *Journal of Ethnic and Migration Studies*, 35(7), pp. 1181–1199.
- Nikulina, V., Widom, C. S. and Czaja, S. (2010) The role of childhood neglect and childhood poverty in predicting mental health, academic achievement and crime in adulthood, *American Journal of Community Psychology*, 48, pp. 309–321.
- Overman, H. G. (2002) Neighbourhood effects in large and small neighbourhoods, *Urban Studies*, 39(1), pp. 117–130.
- Patacchini, E. and Zenou, Y. (2011) Neighborhood effects and parental involvement in the intergenerational transmission of education, *Journal of Regional Science*, 51(5), pp. 987–1013.
- Pattavina, A. (1999) The influence of community violence on child development in an urban setting, *Research in Politics and Society*, 7, pp. 163–182.
- Pong, S. and Hao, L. (2007) Neighborhood and school factors in the school performance of immigrants’ children, *International Migration Review*, 41(1), pp. 206–241.
- Rephann, T. J. (2002) The importance of geographical attributes in the decision to attend college, *Socio*-*Economic Planning Sciences*, 36(4), pp. 291–307.
- Roos, L. L., Hiebert, B., Manivong, P., Edgerton, J., Walld, R., MacWilliam, L. and De Rocquigny, J. (2011) What is most important: Social factors, health selection, and adolescent educational achievement, *Social Indicators Research*, 92(3), pp. 761–781.
- Saatcioglu, A. (2010) Disentangling school- and student-level effects of desegregation and resegregation on the dropout problem in urban high schools: Evidence from the cleveland municipal school district, 1977-1998, *Teachers College Record*, 112(5), pp. 1391–1442.
- Sanson, A., Smart, D. and Misson, S. (2011) Children’s socio-emotional, physical, and cognitive outcomes: Do they share the same drivers? *Australian Journal of Psychology*, 18(3), pp. 311–325.
- Sastry, N. and Pebley, A. R. (2010) Family and neighborhood sources of socioeconomic inequality in children’s achievement, *Demography*, 47(3), pp. 777–800.
- South, S. J., Baumer, E. P. and Lutz, A. (2003) Interpreting community effects on youth educational attainment, *Youth and Society*, 35(1), pp. 3–36.
- Supple, A. J., Ghazarian, S. R., Frabutt, J. M., Plunkett, S. W. and Sands, T. (2006) Contextual influences on Latino adolescent ethnic identity and academic outcomes, *Child Development*, 77(5), pp. 1427–1433.
- Sykes, B. and Kuyper, H. (2009) Neighbourhood effects on youth educational achievement in the Netherlands: Can effects be identified and do they vary by student background characteristics? *Environment and Planning A*, 41(10), pp. 2417–2436.
- Sykes, B. and Musterd, S. (2011) Examining neighbourhood and school effects simultaneously: What does the Dutch evidence show? *Urban Studies*, 48, pp. 1307–1331.
- Tsay, W.-J. (2006) The educational attainment of second-generation mainland Chinese immigrants in Taiwan, *Journal of Population Economics*, 19(4), pp. 749–767.
- Van Der Velden, R. K. W. and Bosker, R. J. (1991) Individuen, gezinnen en buurten: sociale determinanten van het onderwijsniveau [Individuals, families and neighborhoods: Social determinants of educational attainment], *Mens en Maatschappij*, 66(3), pp. 277–295.
- Williams, T. R., Davis, L. E., Saunders, J. and Williams, J. H. (2002) Friends, family, and neighborhood: Understanding academic outcomes of African American youth, *Urban Education*, 37(3), pp. 408–431.
- Wilson, K. (2000) Using the PSID to study the effects of school spending, *Public Finance Review*, 28(5), pp. 428–451.
- Wilson, K. (2001) The determinants of educational attainment: Modeling and estimating the human capital model and education production functions, *Southern Economic Journal*, 67(3), pp. 518–551.
- Witherspoon, D., Schotland, M., Way, N. and Hughes, D. (2009) Connecting the dots: How connectedness to multiple contexts influences the psychological and academic adjustment of urban youth, *Applied Developmental Science*, 13(4), pp. 199–216.
- Wodtke, G. T., Harding, D. J. and Elwert, F. (2011) Neighborhood effects in temporal perspective: The impact of long-term exposure to concentrated disadvantage on high school graduation, *American Sociological Review*, 76(5), pp. 713–736.
- Woolley, M. E. and Grogan-Kaylor, A. (2006) Protective family factors in the context of neighborhood: Promoting positive school outcomes, *Family Relations*, 55(1), pp. 93–104.
- Woolley, M. E., Grogan-Kaylor, A., Gilster, M. E., Karb, R. A., Gant, L. M., Reischl, T. M. and Alaimo, K. (2008) Neighborhood social capital, poor physical conditions, and school achievement, *Children and Schools*, 30(3), pp. 133–145.

## Appendix 2: Descriptive statistics

See Tables 6, 7, 8 and 9.

**Table 6:** Descriptive statistics for the unstandardised coefficients for the neighbourhood poverty model (*N* = 94)

Variable name	Mean	SD	Min.	Max.
Neighbourhood poverty (unweighted)	−0.680	3.330	−29.909	8.88
Location: USA	0.691	0.464	0	1
Location: Europe	0.223	0.419	0	1
Location: other	0.085	0.281	0	1
Sample gender: male	0.096	0.296	0	1
Sample gender: female	0.106	0.310	0	1
Sample gender: mixed	0.798	0.404	0	1
Sample age: 4–10 years	0.191	0.396	0	1
Sample age: 11–20 years	0.649	0.480	0	1
Sample age: 21+ years	0.160	0.368	0	1
Previous educational attainment control variables	0.255	0.438	0	1
Parenting control variables	0.128	0.335	0	1
School-level control variables	0.277	0.450	0	1
Family SES control variables	0.947	0.226	0	1
Sample size (log)	7.748	1.506	5.136	12.475
Use of multilevel	0.330	0.473	0	1
Educ. outcome: high school graduation	0.106	0.310	0	1
Educ. outcome: high school dropout	0.160	0.368	0	1
Educ. outcome: grades/test scores	0.457	0.501	0	1
Educ. outcome: school performance	0.043	0.203	0	1
Educ. outcome: grade retention	0.011	0.103	0	1
Educ. outcome: years of education	0.096	0.296	0	1
Educ. outcome: highest education	0.021	0.145	0	1
Educ. outcome: college attendance	0.085	0.281	0	1
Educ. outcome: college graduation	0.021	0.145	0	1

**Table 7:** Descriptive statistics for the unstandardised coefficients for the poor educational climate model (*N* = 17)

Variable name	Mean	SD	Min.	Max.
Poor educational climate (unweighted)	−1.282	4.852	−20.08	0.544
Location: USA	0.412	0.507	0	1
Location: Europe	0.588	0.507	0	1
Location: other	0.000	0.000	0	0
Sample gender: male	0.059	0.243	0	1
Sample gender: female	0.059	0.243	0	1
Sample gender: mixed	0.882	0.332	0	1
Sample age: 4–10 years	0.000	0.000	0	0
Sample age: 11–20 years	0.471	0.514	0	1
Sample age: 21+ years	0.529	0.514	0	1
Previous educational attainment control variables	0.118	0.332	0	1
Parenting control variables	0.000	0.000	0	0
School-level control variables	0.176	0.393	0	1
Family SES control variables	0.941	0.243	0	1
Sample size (log)	8.469	1.626	6.45	12.475
Use of multilevel	0.176	0.393	0	1
Educ. outcome: high school graduation	0.294	0.470	0	1
Educ. outcome: high school dropout	0.000	0.000	0	0
Educ. outcome: grades/test scores	0.059	0.243	0	1
Educ. outcome: school performance	0.059	0.243	0	1
Educ. outcome: grade retention	0.118	0.332	0	1
Educ. outcome: years of education	0.176	0.393	0	1
Educ. outcome: highest education	0.118	0.332	0	1
Educ. outcome: college attendance	0.176	0.393	0	1
Educ. outcome: college graduation	0.000	0.000	0	0

**Table 8:** Descriptive statistics for the unstandardised coefficients for the proportion of migrant/ethnic groups model (*N* = 48)

Variable name	Mean	SD	Min.	Max.
Proportion ethnic/migrant groups (unweighted)	0.022	4.346	−11.584	23.997
Location: USA	0.750	0.438	0	1
Location: Europe	0.208	0.410	0	1
Location: other	0.042	0.202	0	1
Sample gender: male	0.167	0.377	0	1
Sample gender: female	0.146	0.357	0	1
Sample gender: mixed	0.688	0.468	0	1
Sample age: 4–10 years	0.125	0.334	0	1
Sample age: 11–20 years	0.667	0.476	0	1
Sample age: 21+ years	0.208	0.410	0	1
Previous educational attainment control variables	0.188	0.394	0	1
Parenting control variables	0.063	0.245	0	1
School-level control variables	0.229	0.425	0	1
Family SES control variables	0.958	0.202	0	1
Sample size (log)	7.955	1.570	5.278	12.475
Use of multilevel	0.292	0.459	0	1
Educ. outcome: high school graduation	0.104	0.309	0	1
Educ. outcome: high school dropout	0.125	0.334	0	1
Educ. outcome: grades/test scores	0.375	0.489	0	1
Educ. outcome: school performance	0.042	0.202	0	1
Educ. outcome: grade retention	0.042	0.202	0	1
Educ. outcome: years of education	0.167	0.377	0	1
Educ. outcome: highest education	0.000	0.000	0	0
Educ. outcome: college attendance	0.146	0.357	0	1
Educ. outcome: college graduation	0.000	0.000	0	0

**Table 9:** Descriptive statistics for the unstandardised coefficients for the social disorganisation model (*N* = 47)

Variable name	Mean	SD	Min.	Max.
Social cohesion/order (unweighted)	−2.252	9.050	−61.16	4.41
Location: USA	0.787	0.414	0	1
Location: Europe	0.170	0.380	0	1
Location: other	0.043	0.204	0	1
Sample gender: male	0.021	0.146	0	1
Sample gender: female	0.021	0.146	0	1
Sample gender: mixed	0.957	0.204	0	1
Sample age: 4–10 years	0.319	0.471	0	1
Sample age: 11–20 years	0.489	0.505	0	1
Sample age: 21+ years	0.191	0.398	0	1
Previous educational attainment control variables	0.340	0.479	0	1
Parenting control variables	0.255	0.441	0	1
School-level control variables	0.404	0.496	0	1
Family SES control variables	0.660	0.479	0	1
Sample size (log)	7.226	1.752	4.727	11.397
Use of multilevel	0.383	0.491	0	1
Educ. outcome: high school graduation	0.021	0.146	0	1
Educ. outcome: high school dropout	0.064	0.247	0	1
Educ. outcome: grades/test scores	0.702	0.462	0	1
Educ. outcome: school performance	0.085	0.282	0	1
Educ. outcome: grade retention	0.021	0.146	0	1
Educ. outcome: years of education	0.000	0.000	0	0
Educ. outcome: highest education	0.043	0.204	0	1
Educ. outcome: college attendance	0.064	0.247	0	1
Educ. outcome: college graduation	0.000	0.000	0	0

## Footnotes

1 We also tried to construct a measure for the neighbourhood delineation used in different studies. However, because of the great variety in the used delineations, we were not able to construct a meaningful variable. Furthermore, we tried to include a covariate capturing studies that use techniques to overcome selection bias. However, because these techniques are quite novel and diverse, we were not able to construct this into a workable covariate.
